# Molecular Epidemiology of Bovine Enteroviruses and Genome Characterization of Two Novel Bovine Enterovirus Strains in Guangxi, China

**DOI:** 10.1128/spectrum.03785-22

**Published:** 2023-03-06

**Authors:** Yuhang Luo, Huanghao Liu, Yanlin Zou, Chengpeng Qiao, Yaquan Su, Xinyue Zhu, Guangxin Zhang, Wenfei Huang, Yifeng Qin, Yan Pan, Weijian Huang

**Affiliations:** a Laboratory of Animal Infectious Diseases and Molecular Immunology, College of Animal Science and Technology, Guangxi University, Nanning, China; b Guangxi Agricultural Vocational University, Nanning, China; c Guangxi Zhuang Autonomous Region Engineering Research Center of Veterinary Biologics, Nanning, China; d Guangxi Key Laboratory of Animal Reproduction, Breeding and Disease Control, Nanning, China; e Guangxi Colleges and Universities Key Laboratory of Prevention and Control for Animal Disease, Nanning, China; Institute of Microbiology Chinese Academy of Sciences

**Keywords:** bovine enterovirus, cattle herds, genetic diversity, isolation, recombination, novel enterovirus

## Abstract

Bovine enterovirus (BEV) is a highly infectious pathogen that may cause respiratory and gastrointestinal disease outbreaks in cattle. This study aimed to investigate the prevalence and genetic characteristics of BEVs in Guangxi Province, China. A total of 1,168 fecal samples from 97 different bovine farms were collected between October 2021 and July 2022 in Guangxi Province, China. BEV was confirmed using a reverse transcription-PCR (RT-PCR) method targeting the 5′ untranslated region (UTR), and isolates were genotyped by sequencing their genomes. The nearly complete genome sequences of eight BEV strains showing cytopathic effects in MDBK cells were determined and analyzed. In total, 125 (10.7%) of 1,168 fecal samples were positive for BEV. BEV infection was significantly associated with farming patterns and clinical symptoms (*P* < 0.05; odds ratio [OR] > 1). Molecular characterization indicated that five BEV strains from this study belonged to EV-E2 and one strain to EV-E4. Two BEV strains (GXNN2204 and GXGL2215) could not be assigned to a known type. Strain GXGL2215 showed the closest genetic relationship with GX1901 (GenBank accession number MN607030; China) in its VP1 (67.5%) and P1 (74.7%) and with NGR2017 (MH719217; Nigeria) in its polyprotein (72.0%). It was also close to the EV-E4 strain GXYL2213 from this study when the complete genome (81.7%) was compared. Strain GXNN2204 showed the closest genetic relationship with Ho12 (LC150008; Japan) in the VP1 (66.5%), P1 (71.6%), and polyprotein (73.2%). Genome sequence analysis suggested that strains GXNN2204 and GXGL2215 originated from the genomic recombination of EV-E4 and EV-F3 and EV-E2 and EV-E4, respectively. This study reports the cocirculation of multiple BEV types and the identification of two novel BEV strains in Guangxi, China, and it will provide further insights into the epidemiology and evolution of BEV in China.

**IMPORTANCE** Bovine enterovirus (BEV) is a pathogen that causes intestinal, respiratory, and reproductive disease infections in cattle. This study reports on the widespread prevalence and biological characteristics of the different BEV types which currently exist in Guangxi Province, China. It also provides a reference for the study of the prevalence of BEV in China.

## INTRODUCTION

Enteroviruses (EVs) belong to the genus *Enterovirus* of the family *Picornaviridae*, which are envelope-free icosahedral viruses about 30 to 32 nm in diameter. Each virus particle consists of a 6.7 to 10.1-kb length of unsegmented RNA which contains a unique open reading frame (ORF) (1, 2). All these viruses have similar characteristics, and they are the main pathogens associated with respiratory and gastrointestinal diseases in humans and animals (3). According to the latest classification of enteroviruses, species of bovine enterovirus (BEV) are classified as *Enterovirus-E* (EV-E) and *Enterovirus-F* (EV-F). EV-E consists of five subtypes (E1 to E5), while EV-F has eight (F1 to F8) (4–6).

EV-E and EV-F viruses can be distinguished from other EVs by their unique secondary RNA structures in the 5′-untranslated region (UTR) (domains I* and I**) (1). The ORF encodes a large multimeric protein that is cleaved into four structural and seven nonstructural proteins. The P1 region of the viral polyprotein contains the structural proteins (VP4, VP2, VP3, and VP1), while the P2 and P3 regions contain the nonstructural viral proteins (2A, 2B, and 2C and 3A, 3B, 3C, and 3D, respectively) (7, 8).

BEVs are widespread all over the world due to their rapid transmission via the fecal-oral route. In different parts of the world, BEV is mainly isolated from the feces of cattle, but it can also be isolated from the feces of other animals, including sheep, goats, horses, geese, opossums, and deer (9–12). These viruses have been found in both healthy animals and those with clinical signs of respiratory and enteric diseases, as well as those with fertility disorders. They are also found in the fetal fluids of aborted calves (4, 13).

In addition, BEVs have zoonotic potential, as indicated by the recent demonstration of BEV replication in cells from diverse species, and they have high seroprevalence among humans, horses, dogs, sheep, and goats (14). The pathogenicity and virulence of BEVs remain largely unknown. Previous studies have suggested that they have only limited pathogenicity, as clinical signs of BEV infection have not been successfully replicated in calves (15). As BEV isolates are increasingly identified from cattle, the pathogenicity and virulence of BEVs have gained renewed attention. These studies have increased our understanding of the epidemiology and genetic diversity of BEVs. However, the information on BEVs remains insufficient for the effective control of this viral disease in China.

In the present study, a molecular epidemiological investigation of BEV was conducted in Guangxi Province, China, and the infection rate, infection-related factors, and their genetic characterization were analyzed. Eight strains of BEV were isolated from positive fecal samples, and their genomic characteristics and *in vitro* growth characteristics were also analyzed. This study will promote our understanding of the prevalence and genetic evolution of BEVs in Guangxi Province, China.

## RESULTS

### Prevalence of BEV in Guangxi, China.

**FIG 1:**
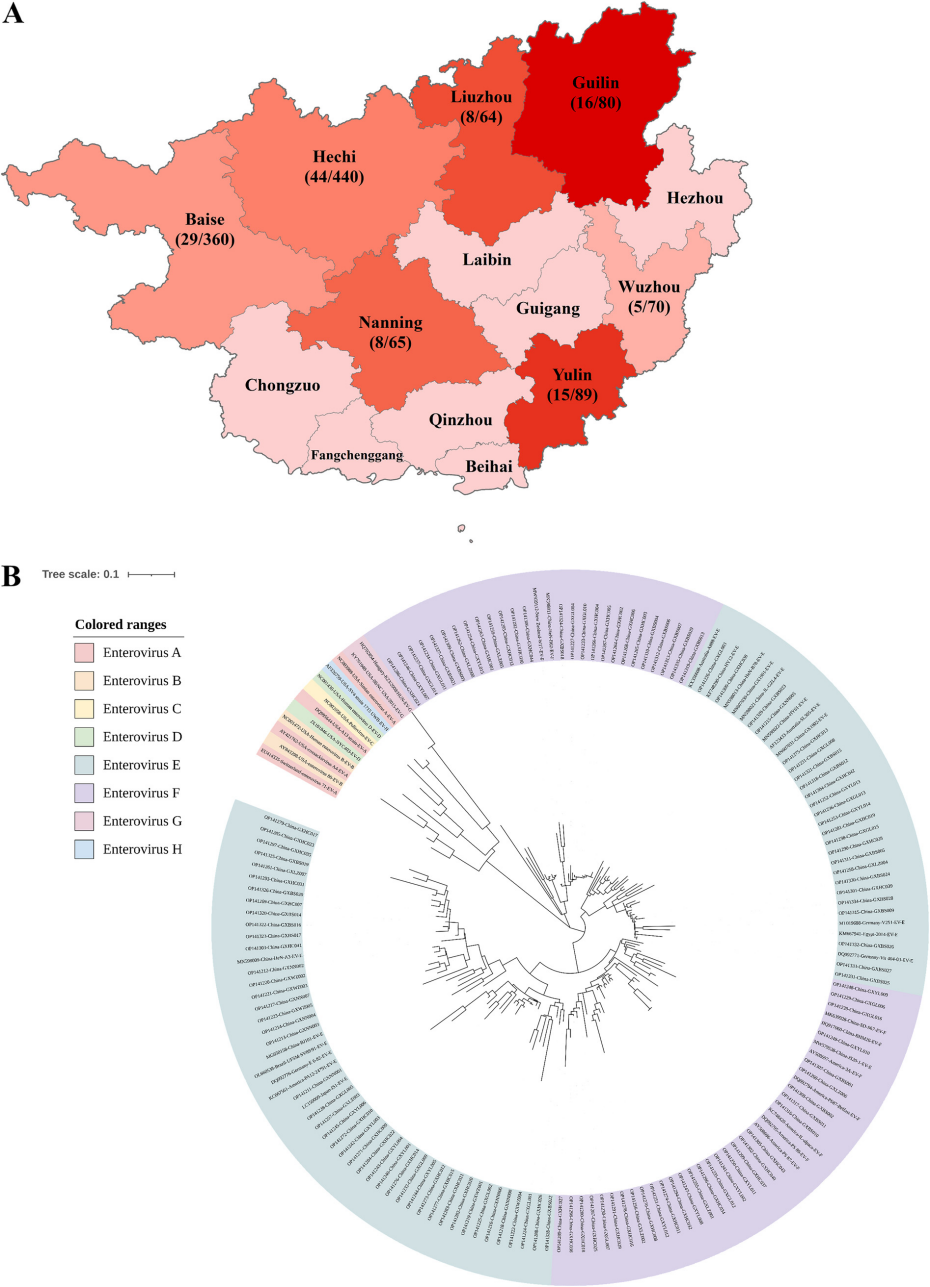
Prevalence and phylogenetic analysis of BEV in Guangxi Province, China. (A) Positivity rates and geographic distribution of BEV in Guangxi Province. (B) Phylogenetic analysis of global BEV strains based on the 5′-UTR gene.

In this study, a total of 1,168 fecal samples were collected from 742 water buffaloes and 426 beef cattle in Guangxi Province, China, from 2021 to 2022. All samples were tested for BEV using reverse transcription-PCR (RT-PCR) targeted to a part of the 5′-UTR gene. This indicated that the total positivity rate for BEV was 10.7% (125/1,168) at the animal level. The positivity rates from highest to lowest in each city of Guangxi Province were as follows: 20.00% (Guilin), 16.86% (Yulin), 12.50% (Liuzhou), 12.31% (Nanning), 10.00% (Hechi), 8.06% (Baise), and 7.14% (Wuzhou) (Fig. 1A). The BEV positivity rate in intensive culture systems was 6.81% (29/426), which was significantly lower than the positivity rate of 12.93% (96/742) in nonintensive culture systems (*P* < 0.05; odds ratio [OR] > 1.000).

Furthermore, the BEV positivity rate in cattle with diarrhea was 27.38% (89/325), which was also significantly higher than the positivity rate of 4.27% (36/823) in cattle without diarrhea symptoms (*P* < 0.05, OR > 1.000) (Table 1). These results indicated that BEV infection was significantly associated with farming patterns and clinical symptoms. There were no significant differences in the BEV infection rates between age (*P* = 0.403) and cattle type (OR < 1.000). The detected BEV-positive sequences were uploaded to the National Center for Biotechnology Information (see “Data availability”). Phylogenetic analysis based on the 5′ UTR showed that 68 and 57 copies of types E and F were detected in this experiment, respectively. These results indicate that BEV infection exists in Guangxi and that both types E and F are prevalent (Fig. 1B).

**TABLE 1:** Correlation analysis of BEV infection

Variable^*a*^	No. of results		*P* value	OR	95% CI^*b*^
	Positive	Negative			
Age					
Calf	73	568	0.403	1.174	0.806–1.710
Adult	52	475			
Farming pattern					
Intensive	29	397	0.001	2.034	1.318–3.139
Nonintensive	96	646			
Cow type					
Water buffalo	61	665	0.001	0.542	0.373–0.787
Native yellow cow	64	378			
Clinical symptoms					
Diarrhea	89	236	0.000	8.454	5.591–12.782
Nondiarrhea	36	807			

a *n* = 1,168.

b 95% CI, 95% confidence interval.

### Isolation and infection characteristics of different BEV strains.

**FIG 2:**
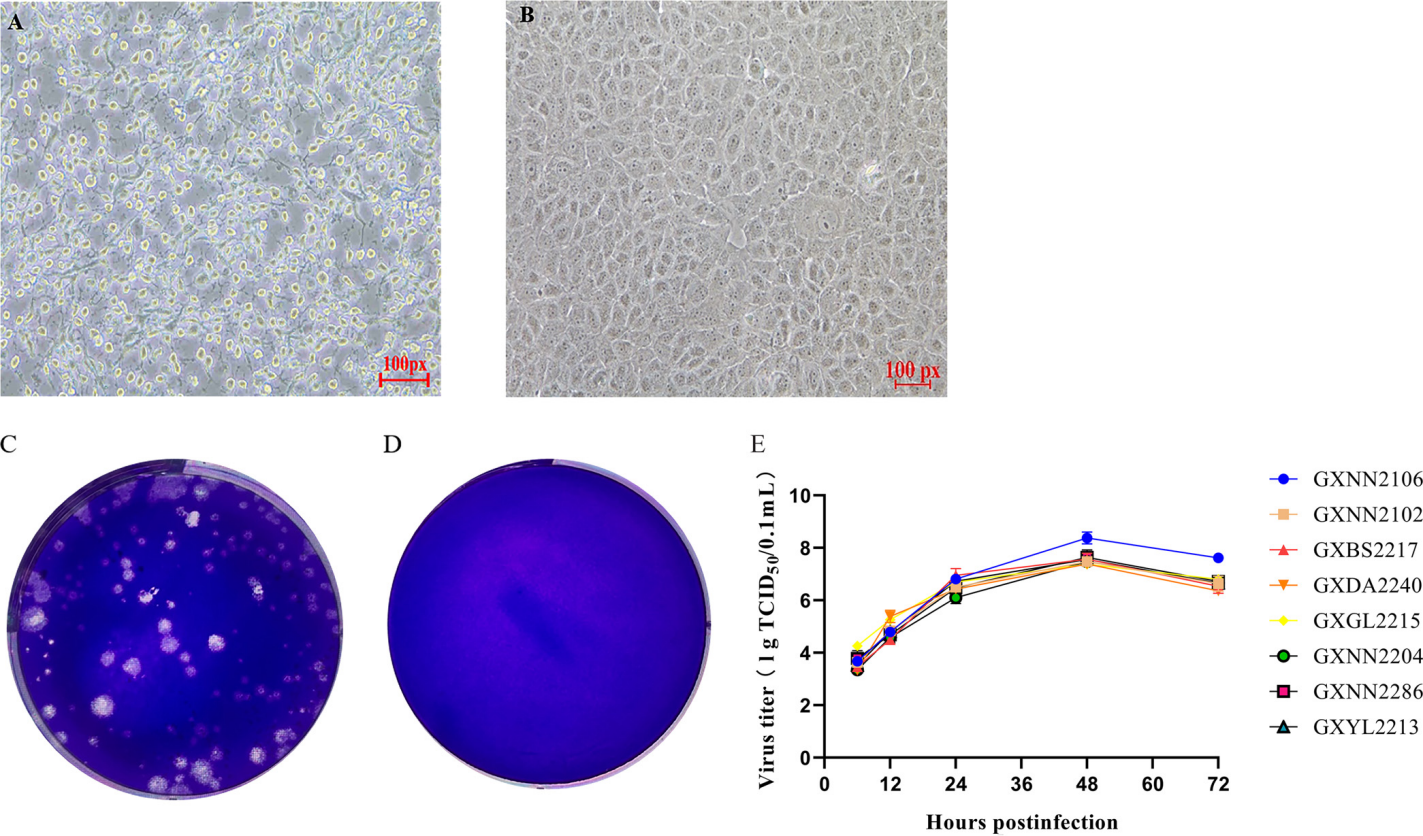
Cytopathogenic effect (CPE), plaque purification, and growth curve analysis in MDBK cells. (A) CPE in MDBK cells infected with strain GXNN2106. Mock-infected and virus-infected MDBK cells were observed at 36 h postinfection (hpi). (B) MDBK cells without virus inoculation (original magnification, ×20). (C) Plaque morphology of strain BEV-GXGL2215 on MDBK cells. (D) Plaque morphology of the mock MDBK cells. (E) Growth kinetics of the eight isolated strains. At different times postinfection, the cell supernatants were collected, and the virus titers were determined as the 50% tissue culture infective dose (TCID_50_) values. The results represented here are the means of three independent experiments.

Fifty fecal samples which were positive for BEV were filtered and used for inoculation into MDBK, PK-15, and Vero cells. Among them, eight samples produced significant cytopathogenic effects (CPEs) in MDBK cells, and these were passed through three consecutive generations. These strains were named GXNN2106, GXNN2102, GXNN2204, GXNN2286, GXGL2215, GXBS2217, GXDA2240, and GXYL2213 (Fig. 2A and B). These BEVs were obtained after a series of plaque purification in MDBK cells. The plaques produced by the BEVs in MDBK cells were round (Fig. 2C and D).

**FIG 3:**
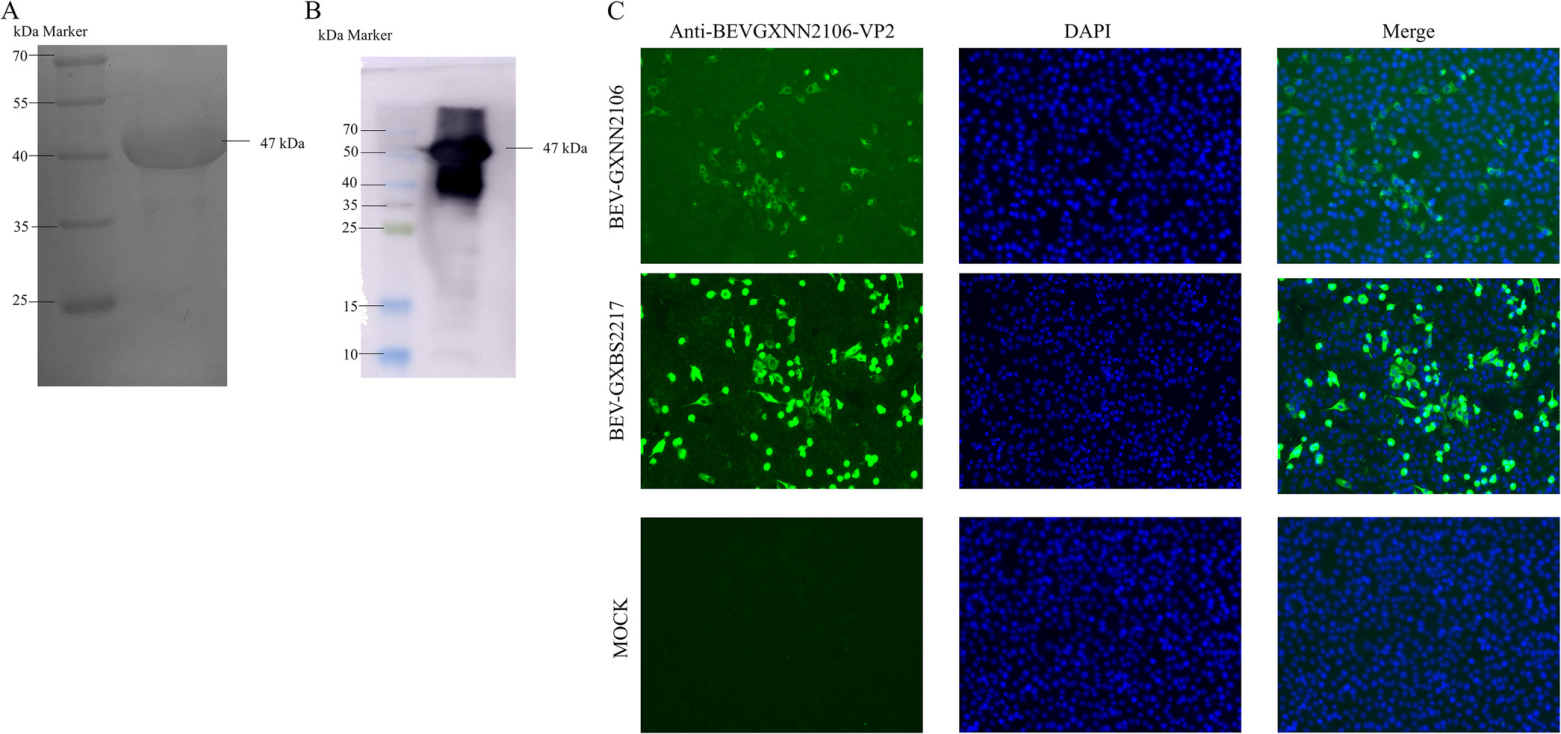
Recombinant protein and IFA analysis. (A) Analysis of expression of the purified BEV-GXNN2106 VP2 by SDS-PAGE. (B) Western blot analysis of expressed recombinant BEV-GXNN2106 VP2 showing reactivity with horseradish peroxidase-conjugated anti-His tag monoclonal antibody. (C) IFA analysis of the VP2 protein expression was conducted in MDBK cells. The virus-infected cells were fixed and stained using an anti-buffalo BEV-VP2 PcAb and goat anti-rabbit IgG H+L (green). The cell nuclei were visualized by DAPI (4′,6-diamidino-2-phenylindole staining) (blue). IFA, indirect immunofluorescence assay.

To generate an antibody against VP2 protein, the VP2 gene of GXNN2106 was cloned into a pET-32a^+^ expression vector, and the resultant recombinant clone was transformed into Escherichia coli cells. The 47-kDa recombinant VP2 protein with a 6×His tag was successfully expressed in the E. coli strain BL21(DE3). The VP2 protein was then purified using a His binding kit, and its molecular weight was confirmed by 12% SDS-PAGE, followed by Western blotting using monoclonal antibodies against the 6×His tag (Fig. 3A and B). Polyclonal antibodies against BEV-VP2 protein were generated by injecting Kunming mice with the purified BEV-VP2 protein.

In order to confirm the isolation of BEV, an indirect immunofluorescence assay (IFA) was conducted using a PcAb raised against BEV-VP2. MDBK cells infected with BEV isolate GXNN2106 were reacted with a specific polyclonal antibody raised against BEV-VP2 (Fig. 3C). Multistage growth curves of the isolated strains were assessed at a multiplicity of infection (MOI) of 0.1. The results (Fig. 2E) showed that the titer of the virus gradually increased starting at 6 h postinfection (hpi) and reached its maximum at 48 hpi. It then slowly decreased, but it remained high at 72 hpi, which indicated that the virus grew well in the cultured MDBK cells.

### Genotypic characterization and diversity of BEV in Guangxi, China.

Eight complete BEV genomes were successfully amplified using six sets of sequence-specific primers. The full lengths of the BEV genomes ranged from 7,404 to 7,618 nucleotides (nt). The genomes contained a large open reading frame (ORF), 3′ UTR, and 5′ UTR. These were compared with full-length genomic sequences of EV-E (E1 to E5), EV-F (F1 to F4), and EV A-J which are available from public databases. The ORF, VP1, and P1 of the eight isolated stains were analyzed, and their homology was compared with different species of enteroviruses. At the nucleotide level, the similarities of the full genomic sequences and polyprotein, VP1, and P1 genes of the 8 strains were 67.1% to 95.5%, 65.5% to 95.5%, 51.7% to 94.7%, and 61.4% to 95.2%, respectively. At the amino acid level, the similarities of the polyprotein, VP1, and P1 were 72.2% to 98.4%, 40.8% to 97.9%, and 61.2% to 98.5%, respectively (Tables 2 and 3). These results indicate large evolutionary distances between the genomes of the isolated strains.

**TABLE 2:** Nucleotide and amino acid sequence identities between strain GXNN2204 and *Enterovirus* reference strains

Reference strain	GenBank accession no.	Identity with GXNN2204 (%)						Subgenotype
		VP1		P1		Polyprotein		
		Nucleotide	Amino acid	Nucleotide	Amino acid	Nucleotide	Amino acid	
PA12-24791	KC667561	62.2	68.8	72.0	88.7	71.7	84.7	EV-E1
NGR 2017	MH719217	63.3	76.0	71.6	84.4	72.0	83.4	EV-E2
HY12	KF748290	63.9	69.9	72.0	83.4	71.6	84.4	EV-E3
GX1901	MN607030	67.5	74.6	74.7	89.1	62.1	66.3	EV-E4
MexKSU/5	KU172420	55.7	53.8	62.6	65.9	67.9	75.7	EV-E5
261	DQ092770	59.2	63.7	62.7	69.4	68.4	77.8	EV-F1
PS 89	DQ092795	60.4	63.6	64.3	68.8	68.7	77.3	EV-F2
Ho12	LC150008	59.7	69.9	63.1	70	68.8	78.4	EV-F3
W1	AY462106	59.8	65.1	63.3	69.4	68.2	76.7	EV-F4
EV71	U22521	49.6	44.4	54.6	52.3	57.1	57.3	EV-A
CA67-10387	AY843298	45.6	38.2	50.8	44.3	57.6	58.1	EV-B
Ca98-10615	DQ995644	44.2	36.4	50.8	46.6	55.5	55.0	EV-C
EV68	NC_038308	48.1	40.8	55.0	51.2	57.2	55.2	EV-D
08/NC	KY761948	57.3	52.30	60.1	63.1	62.8	65.8	EV-G
SV4	AF326759	46.4	44.5	53.8	51.4	57.2	57.1	EV-H
N125	AF414372	49.4	45.7	56.1	54.3	59.2	60.0	EV-J

**TABLE 3:** Nucleotide and amino acid sequence identities between strain GXGL2215 and *Enterovirus* reference strains

Reference strain	GenBank accession no.	Identity with GXGL2215 (%)						Subgenotype
		VP1		P1		Polyprotein		
		Nucleotide	Amino acid	Nucleotide	Amino acid	Nucleotide	Amino acid	
PA12-24791	KC667561	55.1	57.1	62.0	66.9	65.3	72.8	EV-E1
NGR 2017	MH719217	56.8	57.0	62.5	66.4	65.4	72.6	EV-E2
HY12	KF748290	56.8	56.6	62	66.2	65.5	72.4	EV-E3
GX1901	MN607030	57.4	55.1	62.1	66.3	65.7	72.6	EV-E4
MexKSU/5	KU172420	57.1	56.8	62.8	66.1	65.9	72.6	EV-E5
261	DQ092770	62.0	70.1	68.6	79.3	71.8	83.9	EV-F1
PS 89	DQ092795	63.5	68.2	68.4	79.2	71.3	83.5	EV-F2
Ho12	LC150008	66.5	79.9	71.6	85.8	73.2	86.6	EV-F3
W1	AY462106	65.2	75.2	70.3	83.7	71.8	84.0	EV-F4
EV71	U22521	49.8	43.3	54.1	51.2	56.4	56.7	EV-A
CA67-10387	AY843298	44.7	36.6	52.8	44.1	58.5	58.6	EV-B
Ca98-10615	DQ995644	46.3	38.4	52.9	48.9	56.0	55.8	EV-C
EV68	NC_038308	47.5	39.9	54.3	51.5	57.1	55.7	EV-D
08/NC	KY761948	55.0	50.2	59.4	60.5	62.6	65.5	EV-G
SV4	AF326759	47.2	45.8	53.8	54.1	57.6	58.1	EV-H
N125	AF414372	50.8	44.0	55.7	55.5	59.3	60.4	EV-J

**FIG 4:**
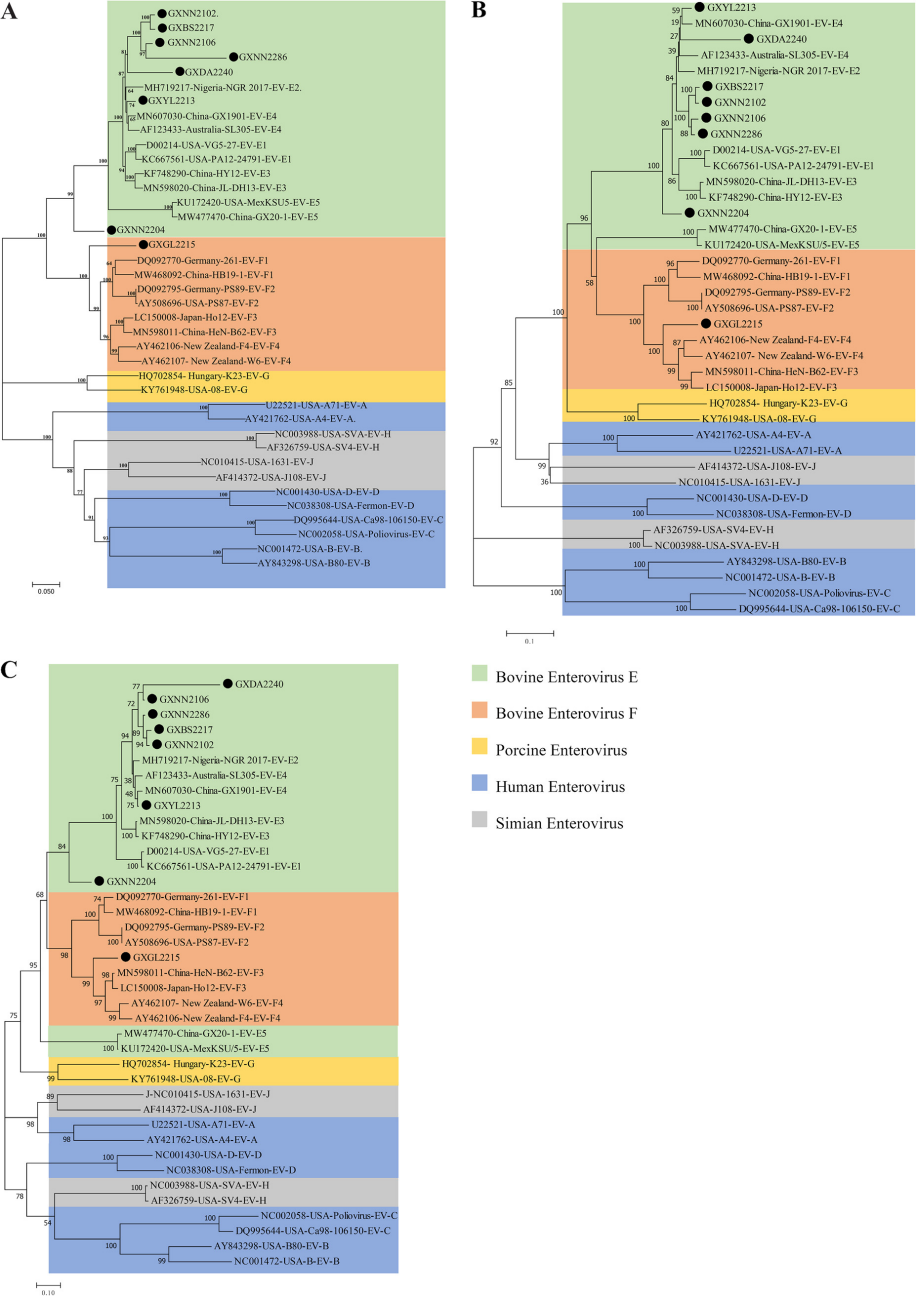
Phylogenetic trees based on the full-length amino acid sequences of the polyprotein (A), P1 (B), and VP1 (C) genes in the virulent strains isolated (black dots).

Upon phylogenetic analysis based on the polyprotein, VP1, and P1 amino acid sequences (Fig. 4A to C), strains GXNN2204 and GXGL2215 were assigned to independent branches in types E and F, respectively. The five E-type BEV isolates (GXNN2102, GXBS2217, GXNN2106, GXDA2240, and GXNN2286) were related genetically to the subtype BEV-E2 isolate NGR 2017 (GenBank accession number MH719217), from Nigeria. GXYL2213 was clustered with the subtype BEV-E4 isolates previously isolated in Guangxi, including GX1901 (MN607030), and shared higher amino acid identities with them, with an average of 96.7%. This suggests that the BEV-E4 subtype has long been circulating among bovine populations in Guangxi.

Significantly, strains GXNN2204 and GXGL2215 are in separate branches on each tree of subtypes E and F, respectively. In the VP1, P1, and polyprotein trees, two strains failed to cluster with EV-E or EV-F. Our phylogenetic tree shows that these two strains form a separate branch but fail to cluster with any other previous bovine enteroviruses. These results suggest that GXNN2204 and GXGL2215 are taxonomically distant from previously reported BEVs, based on taxonomic definitions. We decided to classify these two strains as two new phylogenetic types of enteroviruses.

### Recombination analysis of isolated strains to explore the potential evolutionary process of enteroviruses.

**FIG 5:**
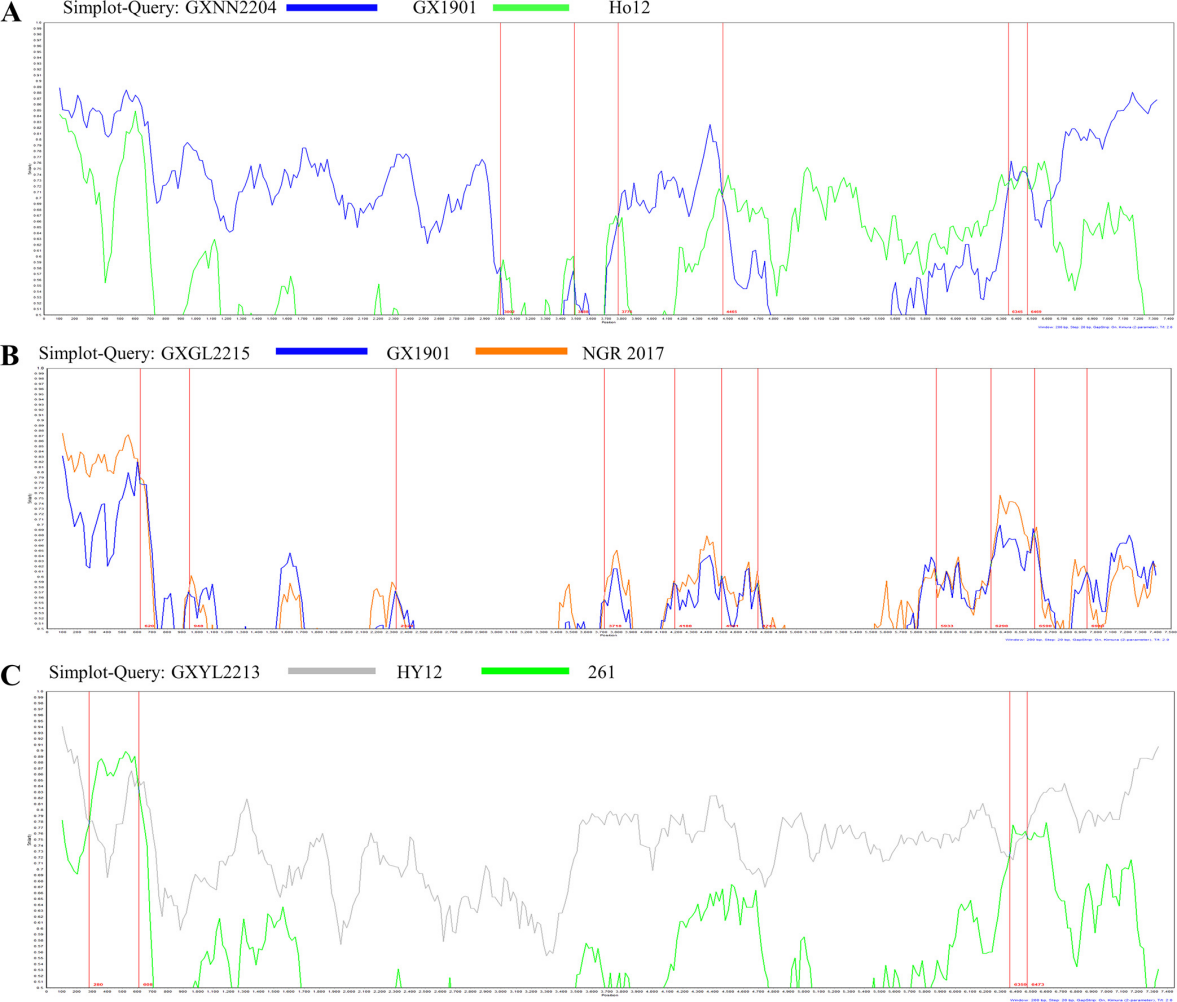
Recombination within the genomes of the virulent strains GXYL2213 (A), GXNN2204 (B), and GXGL2215 (C). Recombination analysis performed using RDP5.

The recombination events of the eight isolated BEV strains were analyzed using the RDP5 and Simplot software packages (Table 4 and Fig. 5A to C). The results showed that three strains had occurred through gene recombination. Among these, strain GXNN2204 was a recombinant of BEV-F3 strain Ho12 (GenBank accession number LC150008; Japan) and the BEV-E4 strain GX1901 (MN607030; China). A similarity plot analysis showed that the genome had seven recombination breakpoints (positions of alignment), located in the VP1 (nt 3002), 2A (nt 3488 and nt 3776), 2C (nt 4465), and 3D (nt 6345 and 6469) regions (Fig. 5A).

**TABLE 4:** Information on recombination events of BEV isolates detected using RDP5 software

Strain	Parental sequences		*P* value for detection method:						
	Minor	Major	RDP	GENECONV	Bootscan	MaxChi	Chimaera	SiScan	3Seq
GXYL2213	261	HY12	5.981 × 10^−13^	1.663 × 10^−2^	1.951 × 10^−15^	3.954 × 10^−8^	3.187 × 10^−9^	1.686 × 10^−6^	7.960 × 10^−8^
GXNN2204	GX1901	Ho12	1.246 × 10^−17^	1.023 × 10^−3^	6.348 × 10^−10^	5.317 × 10^−267^	2.097 × 10^−21^	8.593 × 10^−30^	3.167 × 10^−7^
GXGL2215	GX1901	NGR 2017	5.918 × 10^−9^	1.470 × 10^−2^	6.143 × 10^−8^	1.495 × 10^−5^	1.009 × 10^−7^		2.740 × 10^−9^

Strain GXGL2215 was a recombinant of the BEV-E2 strain NGR2017 (GenBank accession number MH719217; Nigeria) and the BEV-E4 strain GX1901 (MN607030; China). A similarity plot analysis showed a high number of GXGL2215 breakpoint locations, with 11 recombination breakpoints in the genome (positions of alignment), located in the 5′ UTR (nt 620) and the VP4 (nt 948), VP3 (nt 2320), 2A (nt 3718), 2C (nt 4188, nt 4561, and nt 4743), 3C (nt 5933), and 3D (nt 6298, nt 6590, and nt 6940) regions (Fig. 5B).

Strain GXYL2213 was a recombinant of the BEV-E3 strain HY12 (GenBank accession number KF748290; China) and the BEV-F1 strain 261 (DQ092770; Germany). A similarity plot analysis showed that the genome had four recombination breakpoints (positions of alignment), located in the 5′ UTR (nt 280 and nt 608) and 3D region (nt 6359 and nt 6473) (Fig. 5C).

The results of the software analysis showed that recombination events were most likely to occur in the 5′ UTR and 2A, 2C, and 3D regions.

## DISCUSSION

BEV is one of the most important pathogens causing digestive, respiratory, and reproductive tract diseases in cattle, which can be readily coinfected with BEV and other viruses, causing significant economic losses in the cattle industry (16, 17). The prevalence of BEV has been reported worldwide, with positivity rates of 67% in Thailand, 14.5% in Brazil, 78% in Spain, 22.83% in Turkey, and 21.49% in Henan Province, China (10, 14, 18–20). However, so far, there have been no reports on the prevalence of BEVs in Guangxi Province, China, which is one of the main cattle and buffalo farming regions.

In this study, 1,168 fecal samples from cattle in different cities and counties in Guangxi were tested using primers designed to be BEV 5′ UTR specific. The results showed that the BEV positivity rate in Guangxi was 10.7%, lower than that reported in other places. We suspect that this result may be related to the limitations of the primers we designed. This was discussed by Baoming Liu (21) in a previous study in which universal primers were used for the detection of enteroviruses. This prompted us to use 5′-UTR primers that were designed specifically for the Guangxi strain. Statistical analysis showed that the BEV positivity rate in Guangxi was significantly related to farming patterns and diarrhea in the farmed animals. The BEV positivity rate was significantly higher in cattle with free-range farming patterns; this finding may be related to factors such as whether vaccination was given on time and whether attention was paid to biological control. In addition, the BEV positivity rate in diarrheic cattle was significantly higher than that in healthy cattle, suggesting that BEV is an important cause of diarrhea in cattle, which is consistent with previously reported results (4, 13).

However, we also detected a 4.27% BEV positivity rate in fecal samples from clinically healthy cattle. This indicates that BEV can also cause asymptomatic infections in cattle; infected cattle can excrete the virus to the outside world (1), and this can lead to the spread of BEVs throughout the herd. This is a potentially dangerous source of infection. Therefore, it is important to increase the surveillance of BEV infections in cattle to avoid large-scale BEV epidemic outbreaks in the future.

Genetic evolutionary analysis of the BEV 5′ UTR revealed that among the 125 positive samples we obtained, 54.4% and 45.6% belonged to the BEV infection types E and F, respectively. BEV is currently divided into 13 subtypes, and the differences between the subtypes have not been studied in detail. In the evolutionary tree analysis, there were also different clusters of branches of types E and F. This indicates that there are large differences in the presence of BEV in different cities and counties in Guangxi and that there are different subtypes of BEV infection. Combining this information with the BEV infections reported around the world (15, 21–23), we recommend strengthening BEV surveillance for factors related to the introduction of new cattle, as well as the import of beef and milk-based products.

In this study, eight strains of BEV were isolated, and genetic evolutionary analysis revealed that seven of these belonged to BEV type E. Among them, four strains, GXNN2106, GXNN2102, GXNN2286, and GXDA2240, were subtype BEV-E2, and GXYL2204 was subtype BEV-E4.

According to International Committee on Taxonomy of Viruses (ICTV) classification criteria (amino acid identities of >70% in polyproteins and >60% in P1), GXNN2204 and GXGL2215 have 66% to 89% sequence identity with other BEVs in their P1 protein and 72% to 86% identity in their polyproteins. Therefore, a separate branch was formed in all phylogenetic analyses, including VP1, P1, and polyprotein, suggesting that GXNN2204 and GXGL2215 constituted two new genotypes in the genus *Bovine enterovirus* (24).

The homologies between the eight isolated strains in the polyprotein, VP1, and P1 amino acid sequences were 72.2% to 98.4%, 40.8% to 97.9%, and 61.2% to 98.5%, respectively, indicating a large genetic difference between these isolates. The multistep growth curves of these eight isolates showed that they were all proliferated stably in MDBK cells, but we have not yet modeled the growth of the individual viruses in the gut; thus, their growth characteristics in the gut are unknown to us.

There are a few reports on recombination analysis in BEV. In this study, the RDP5 and Simplot software packages were used to assess the possible recombination events in the eight isolated strains, and recombination was found in three of them. Strains GXNN2204 and GXGL2215 shared the parental subtype BEV-E4 strain GX1901 (GenBank accession number MN607030), which was previously isolated in Guangxi. This suggests that some local endemic strains may have undergone genetic recombination with some exotic strains, leading to the emergence of new strains. This is similar to the possible viral recombination of GX20-1 reported recently by Ji et al. upon isolation of BEV-E5 (20). In this regard, we speculate that strain GX1901 (MN607030) played an important role in the epidemic spread of BEV in Guangxi for the production of recombinant fragments with other viruses. Whether the BEV-E4 subtype continues to be endemic in Guangxi needs to be further strengthened by surveillance.

In summary, we conducted an epidemiological survey of BEV in Guangxi, China, and showed that BEV is widely spread in Guangxi and that BEV infection is significantly associated with farming patterns and clinical diarrhea conditions. Phylogenetic analysis showed that there were multiple strains of types E and F circulating in Guangxi. Eight strains of BEV were isolated from fecal samples using cultured MDBK cells. By studying the whole-genome sequences and viral culture characteristics of the viruses, we revealed the molecular and viral biology of different BEV subtypes in Guangxi, China. Our study will provide a reference for understanding the molecular epidemiological characteristics of BEVs in China.

## MATERIALS AND METHODS

### Sample collection and detection.

A total of 1,168 fecal samples were collected from 97 different bovine farms in Guangxi Province, China, from October 2021 to July 2022 (Table 1). All the samples were shipped on ice and then stored at −80°C. The samples were treated as described previously (16). Briefly, the samples were diluted with Dulbecco’s phosphate-buffered saline (DPBS) containing an antibiotic/antimycotic solution. The diluted samples were frozen and thawed 3 times, followed by centrifugation at 10,000 × *g* at 4°C for 10 min. Aliquots (1 mL) of the fecal supernatants were collected and stored at −40°C for RNA extraction and virus isolation.

Viral nucleic acid was extracted using an RNA extraction kit (Axygen, Inc., USA) according to the manufacturer’s instructions. RT-PCR was then performed to detect BEV using the following primers: BEV-F (5′-CCGACTCCGCACCGATACGTCG-3′) and BEV-R (5′-CTCTCAGAGCTACCACTGGGGT-3′). The thermal cycling conditions for each PCR fragment amplification were as follows: predenaturation at 98°C for 2 min, followed by 35 cycles of 95°C for 30 s, 59°C for 30 s, and 72°C for 30 s, and a final elongation step at 72°C for 10 min. Negative controls were set up to monitor cross-contamination during the handling of disease materials. Controls were also used during the sampling and monitoring of RT-PCR. PCR products were subjected to electrophoresis in 1.5% agarose gels, visualized with UV light (Bio-Rad, Inc., USA) and/or purified for DNA sequencing (Sangon Biotech, Inc., China). The sequences were compared with existing sequences in publicly available databases using the Basic Local Alignment Search Tool (BLAST).

### Cell culture.

Madin-Darby bovine kidney cells (MDBK; ATCC CCL-22) were maintained in Dulbecco’s modified Eagle’s medium (DMEM; Gibco, Inc., USA) supplemented with 10% fetal bovine serum (FBS; Gibco, Inc.) at 37°C in a 5% CO_2_-enriched atmosphere.

### Virus isolation and identification.

The BEV-positive clinical samples were homogenized in DPBS containing 200 U/mL penicillin, 200 mg/mL streptomycin, and 100 μg/mL gentamicin. They were then centrifuged at 10,000 × *g* for 5 min at 4°C. The supernatants were filtered through 0.22-μM filters (Millipore, Inc., USA) and added to MDBK cells. After observation at 6, 12, 24, and 48 h postinfection, passaging continued until obvious cell pathogenic effects (CPEs) appeared. The cell supernatants were then used to extract total RNA, and the full-length BEV genome was amplified.

### Primer design and amplification of the full-length genome.

The BEV full-length genome is divided into six overlapping fragments of ~1.5 kb (nt 1 to 1538), ~1.6 kb (nt 1351 to 2948), ~1.5 kb (nt 2602 to 4169), ~1.1 kb (nt 3863 to 5002), ~1.3 kb (nt 4694 to 6007), and ~1.9 kb (nt 5592 to 7500). These fragments were amplified using six corresponding sets of primers (Table 5). All of the primers were designed to enable sequencing of the entire segments, with ~200-nt overlaps between them. The PCR was performed in a 50-μL reaction mixture containing 6 μL of template cDNA, 25 μL of PrimeSTAR Max DNA polymerase (TaKaRa Bio, Inc., Japan), and 1 μL of each forward and reverse primer, with double-distilled water (ddH_2_O) added to reach a total volume of 50 μL. PCR was performed under the following conditions: a predenaturation step at 98°C for 2 min, 30 cycles of denaturation at 98°C for 15 s, annealing at 55°C for 15 s, and extension at 72°C for 25 s, followed by a final extension at 72°C for 10 min. Then, the PCR products were purified using an E.A.N.A. gel extraction kit (Tiangen, Inc., China) and subjected to Sanger sequencing (Sangon Bio, Inc.). The purified PCR products were sequenced from both the 5′ to 3′ and 3′ to 5′ directions to ensure that no mutations arose in these segments. Each segment was sequenced a minimum of two times. The amplified and spliced full sequences were uploaded to GenBank (see “Data availability”).

**TABLE 5:** Primers used to amplify the full-length genomes of eight bovine enteroviruses

Primer name^*a*^	Primer sequence (5′–3′)	Genomic position (nt)	Length of PCR product (bp)
BEV-1F	TTTAAAACARCCTGGGG	1–1538	1,538
BEV-1R	CAGGGCATTWCCCAGGC		
BEV-2F	CAGTGCAARGCCACCAAG	1351–2948	1,597
BEV-2R	GGTGGAYCATACATGCA		
BEV-3F	GAAACBGGWGCCACGTC	2602–4169	1,567
BEV-3R	GCTGCAATCCAATCRAG		
BEV-4F	CTSAGGGACATGCTTG	3863–5002	1,139
BEV-4R	GACTGTACCMCATRCCA		
BEV-5F	GTMCCCKTGGCTGCCC	4694–6007	1,313
BEV-5R	CCTTYCTCTTTTCAATG		
BEV-6F	GCCTARGATTTGAATGA	5592–7500	1,908
BEV-6R	ACACCCCATRCGGTGGGTGTATT		

a F and R represent the upstream and downstream primers, respectively.

### Plaque assay and purification.

After thrice freezing and thawing the virus cultures, the supernatants were removed by centrifugation at 10,000 × g for 30 min at 4°C. Viral plaque assays were performed using MDBK cells seeded in 6-well plates. MDBK cells were inoculated with 10-fold serially diluted viral samples and incubated at 37°C for 1 h. The cells were overlaid with a mixture of DMEM containing 1% low-melting agarose (Cambrex, Inc., USA) and 2% FBS and incubated for 3 days at 37°C in an atmosphere of 5% CO_2_. The medium was then carefully removed from the plates, and the cells were stained with 3 to 4 mL of staining solution, which comprised 0.5% crystal violet and 25% formaldehyde solution, for 15 min. To obtain a single viral clone, the viral plaques were purified. Three viral plaques in the agarose were selected using pipettes and dispersed in DMEM. After centrifugation, the supernatants were used to infect MDBK cells. The harvested viruses were serially passaged on MDBK cells. The above-described experimental steps were repeated 3 times to obtain the purified virus.

### TCID_50_ titration and growth curves of virus isolates.

Titration of the 50% tissue culture infective dose (TCID_50_) for the isolates was performed using 96-well plates. Briefly, the viruses were diluted at 10× serial dilutions and used to infect the wells at each dilution. Then, 72 h postinoculation, the CPEs on the MDBK cells were observed and counted, and the TCID_50_ was calculated following a standard procedure. The virus titers (TCID_50_) for each time point were assessed and calculated based on the Reed-Muench method (25) to describe the viral growth kinetics. The virus isolates (P5) were briefly inoculated into the MDBK cells growing in the six-well plates at an MOI of 0.01. After incubation at 37°C for 1 h, the cells were washed twice with PBS. MDBK cell supernatants (200 μL) were harvested at 6, 12, 24, 48, and 72 hpi and stored at −80°C. The growth curves were determined by measuring the mean titers of three independent measurements at each time point.

### Antibody, SDS-PAGE, and Western blot analysis.

To generate an antibody against VP2 protein, the VP2 gene of BEV-GXNN2106 was amplified by RT-PCR and cloned into the pET-32a^+^ expression vector (Novagen, Inc., Germany), resulting in plasmid pET32a-VP2. pET32a-VP2 was transformed into Escherichia coli BL21(DE3) cells. The cells were then induced by 0.1 mM IPTG (isopropyl-β-d-thiogalactopyranoside) for 6 h. The recombinant protein was purified using a His binding kit (Novagen, Inc.).

Polyclonal antibodies raised against the BEV-VP2 protein were generated by injecting Kunming mice with the purified BEV-VP2 protein. This polyclonal antibody was purified by affinity chromatography with protein A. Protein lysates were subjected to 12% SDS-PAGE, and the proteins were transferred to a polyvinylidene fluoride (PVDF) membrane (Millipore, Inc.). The PVDF membranes were blocked with 5% nonfat dry milk for 2 h at 37°C. They were then incubated with either monoclonal antibodies against the 6×-His tag or anti-BEV-VP2 PcAb overnight at 4°C. Subsequently, the membranes were washed five times with Tris-buffered saline Tween 20 (TBST) and incubated with goat anti-mouse IgG horseradish peroxidase (HRP) secondary antibody conjugate (H+L; 1:5,000; Abmart, Inc., China) for 1 h at 37°C. After five washes with TBST, the membrane-immobilized proteins were visualized using an enhanced chemiluminescence detection system.

### Indirect immunofluorescence assay.

MDBK cells were inoculated with BEV-GXNN2106 and BEV-GXBS2217. At 48 hpi, the inoculum was discarded. The cell monolayers were washed three times with PBS and then fixed with cold acetone at −20°C for 30 min. After five washes with PBS, the cells were incubated with the primary antibody, anti-BEV-VP2 PcAb (1:200), for 2 h at 37°C. After five more washes with PBS, the cells were incubated with goat anti-mouse IgG H+L (Alexa Fluor 488; Proteintech, Inc, USA) secondary antibody, for 1 h at 37°C. Following five washes with PBS, the cell nuclei were stained with DAPI (4′,6-diamidino-2-phenylindole; Solarbio, Inc., China) for 5 min. Finally, the cells were observed under a fluorescence microscope.

### Sequence analysis.

To determine the genetic characteristics of BEV, evolutionary and phylogenetic analyses were focused on the 5′ UTR genes of 58 BEV strains from different countries available at GenBank on 29 August 2022. The genome sequences and deduced amino acid sequences of the 8 strains were aligned and analyzed using the programs SeqMan and MegAlign (DNASTAR, Madison, USA). Multiple alignments were performed between these strains and the 58 genomics found in the GenBank database. Alternative models were constructed for maximum likelihood (ML) phylogenetic tree analysis to test out the optimal analysis methods and models. Phylogenetic trees of the polyprotein, VP1, and P1 amino acid sequences were constructed using the ML method with the Tamura-Nei and Kimura 2 parameter models, respectively. This was performed using MEGA X software (http://www.megasoftware.net/) and 1,000 bootstrap values. The putative recombinant origin of the 30 BEV genome sequences (including the 8 isolates in this study) was determined using a recombination detection software package (RDP5.3). Seven different methods were employed, including RDP, GENECONV, MaxChi, Bootscan, SiScan, and 3Seq, with default parameters, and the *P* values obtained were expected to satisfy at least six of the algorithms (*P* ≤ 10^−6^). The recombinant events in the BEV genomes were confirmed by analysis using Simplot v3.5.1 software (JHK University, Baltimore, MD, USA) with default parameters.

### Data availability.

The BEV isolates were uploaded to the National Center for Biotechnology Information under GenBank accession numbers OP141211 to OP141335. The amplified and spliced full sequences were uploaded to GenBank under accession numbers OL630964 (GXNN2106), OM912604 (GXNN2102), ON457567 (GXNN2286), ON677252 (GXBS2217), ON457566 (GXDA2240), ON630762 (GXGL2215), and ON584772 (GXYL2213).
